# Anatomic safeguards and the low risk of vascular injury during percutaneous tricortical S1 pedicle screw fixation – a cadaver study

**DOI:** 10.1038/s41598-026-38331-y

**Published:** 2026-02-03

**Authors:** Katharina Koeck, Lukas F. Reissig, Andreas Hainfellner, Wolfgang J. Weninger, Stefan Grossauer

**Affiliations:** 1 Center of Anatomy and Cell Biology, Medical University of Vienna, Vienna, Austria; 2 Vienna Medical Center, Vienna, Austria

**Keywords:** Percutaneous tricortical S1 pedicle screw fixation, Vascular injury, Anatomy, Cadaver study, Screw abutment, Sacral spine, Translational research, Anatomy, Musculoskeletal system

## Abstract

Percutaneous tricortical S1 pedicle screw fixation (PTSPSF) offers superior fixation compared to bicortical or monocortical methods but poses potential risks to major intrapelvic vessels. Strangely, clinical complications are rare. This study aims to elucidate the reasons for this discrepancy. In 17 fresh human cadavers, 34 pedicle screws were placed under fluoroscopic guidance, ensuring the distal two threads to exceed the anterior cortical promontory wall. 12 specimens were turned supine and anatomically dissected. The relation between the sacral promontory tip, screw tips and intrapelvic vessels was carefully examined. In 5 specimen 3D fluoroscopy was conducted to measure the distance between the screw tips and the surrounding blood vessels. No intraluminal screw placements were observed. In 2 cases (8.3%), screw tips penetrated the anterior longitudinal ligament and abutted the right common iliac vein (CIV), but without causing impression or injury of the vessel wall. In 2 other cases the ligament remained intact and the screw trajectory pointed dorsally to the CIV. Again the wall of this vessels remained intact. In 84%, the screws were surrounded by osteophytes and did not touch or point to major vessels. Osteophytes at the sacral promontory are typically not visible during lateral fluoroscopy-guided surgery, yet they are commonly present in older individuals. As demonstrated in our study, screw trajectory plays a critical role in avoiding vascular injury, with osteophytes potentially offering an added layer of protection to the right common iliac vein (CIV) and other major vessels. In a post-mortem setting, our findings suggest that even when screw tips penetrate the anterior longitudinal ligament and extend two threads beyond the bone, there is no direct injury to the walls of major blood vessels.

## Introduction

Lumbosacral fixation is the preferred treatment for addressing instability of the lumbosacral vertebral column. Among available surgical options, posterior transpedicular sacral screw fixation has emerged as the gold standard. Achieving rigid screw fixation in the sacral pedicles typically requires the screw tips to penetrate either the anterior sacral wall (bicortical fixation) or the apex of the sacral promontory (tricortical fixation). Although tricortical fixation provides optimal outcomes^1,2^, it also carries an increased risk of neurovascular injury^3–5^​. Yet, astonishingly, the overall complication rate remains low, with an incidence of less than 0.16% in open surgeries​^6^ and 0.13% in percutaneous pedicle screw fixation​^7^.

In a retrospective review of the frequency of intraoperative vessel injury in 182 patients who underwent thoracolumbar and lumbosacral pedicle screw fusion through a standard midline open approach postoperative imaging revealed that 33 of the 680 screws placed were in contact with major vessels; the majority (22) with the iliac veins. However, none of these vessels showed deformation due to screw contact​^8^. Yet, this study had to purely rely on radiologic imaging, and they had no possibility to perform anatomic examinations to investigate the reason for this phenomenon.

Our cadaver study aims to explore the anatomy of the pelvis of body donors who received fluorescence-guided percutaneous tricortical application of sacral (S1) pedicle screws. It focuses on the relation between the major blood vessels and the pedicle screws to explore the reasons for the relatively low risk of neurovascular injury during such interventions and to contribute to the safety profile.

## Materials and methods

### Surgical procedure and specimen preparation

The study utilized 17 fresh human cadavers (8 female, 9 male) with no history of sacral spinal surgery. Their average age was 85 years, ranging from 65 to 97 years. All specimens were obtained from body donors who had voluntarily provided written informed consent during their lifetime for the donation of their bodies for teaching and scientific research. All procedures were conducted in accordance with institutional guidelines and applicable regulations.

The study was approved by the Ethics Committee of the Medical University of Vienna, Austria (approval number 1551/2023). All procedures adhered to the tenets of the Declaration of Helsinki.

**Fig. 1:**
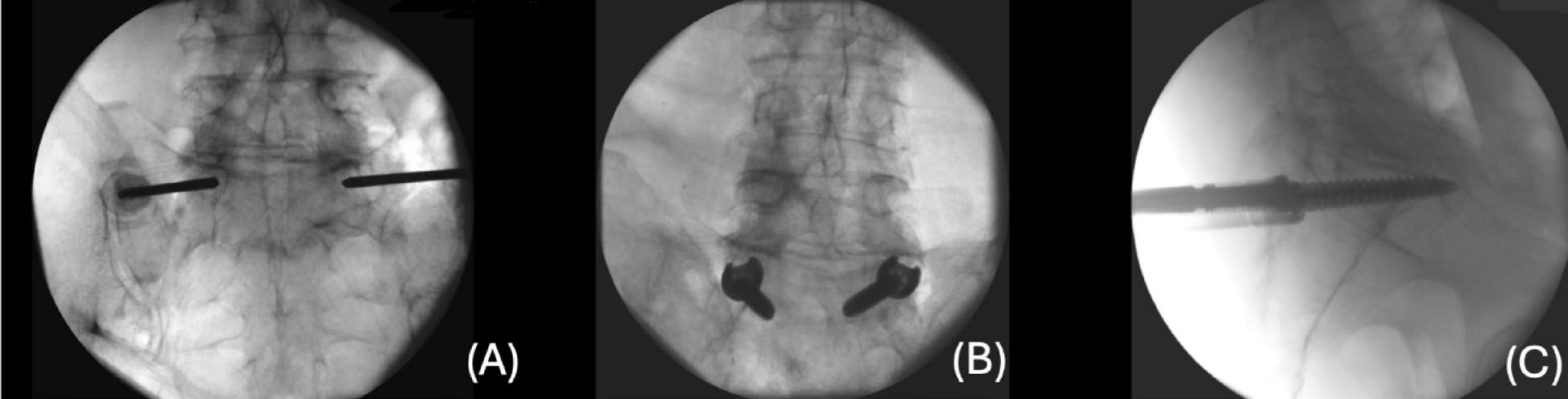
Intraoperative fluoroscopy of percutaneous tricortical S1 pedicle screw fixation. (**A**) Bilateral Jamshidi needle. Placement demonstrated by an a.p. radiograph, (**B**) a.p. radiograph confirming correct positions of the pedicle screws. (**C**) Anterior sacral cortex, exceeded by two threads of the pedicle screw tips. Lateral radiograph.

Cadavers were placed in a prone position with a single C-arm (Cios Spin, Siemens) positioned laterally on the right side. Ferguson anterior-posterior fluoroscopy was employed to localize the pedicle and identify the entry point. The gantry alignment was adjusted to align with the cranial endplate of the S1 vertebra, then rotated to center the spinous process between the pedicles. Bilateral Jamshidi needles, which are hollow stainless-steel needles with a sharp beveled tip, were inserted at the 4 o’clock (right side) and 8 o’clock (left side) positions to prepare pathways for screw placement. Serial anterior-posterior and lateral radiographs documented the progression of the needles (Fig. 1a, b). Lateral radiographs confirmed the screw tips were aligned within the midline of the pedicles. After successful cannulation, a K-wire was guided through the Jamshidi needle into the vertebral body, avoiding penetration of the promontory’s anterior cortex. A 5.5 mm tap and 5.5 mm polyaxial cannulated pedicle screws were inserted over the guide wire, with both screws positioned two threads anterior to the cortex at the sacral promontory tip (Fig. 1c). All procedures were performed by an experienced spine surgeon, using the Stryker ES2 MIS pedicle screw system.

#### Screw trajectory

S1 screws were inserted parallel to the S1 superior endplate with ~ 20° medial convergence toward the apex of the promontory, verified under fluoroscopy; tips were limited to two threads beyond the anterior cortex. This angle was selected to optimize screw purchase while minimizing the risk of neurovascular compromise.

Upon completion of the procedure 3D imaging were conducted on the implanted screws in seven cadavers to verify the screw angles and to measure the distance between the screw tip and the surrounding blood vessels.

12 of the total 17 specimen were repositioned supine, and an abdominal dissection was conducted. Abdominal and pelvic organs were exposed, retracted, and carefully inspected for injury. The sacrum, the prevertebral neurovascular structures and the sacral screw tips were carefully exposed and macroscopically analyzed without changing their relations. Finally, the blood vessels touched by screw tips were removed and their walls were carefully inspected.

### Statistical analysis

Descriptive statistics was performed by using the Excel software package. (MS Excel for Mac, Version 16.89.1)

## Results

In 100% of specimens, the visceral organs, as well as the bifurcation of the aorta and inferior vena cava—which were consistently positioned cranially to the sacral promontory—remained unharmed.

**Fig. 2:**
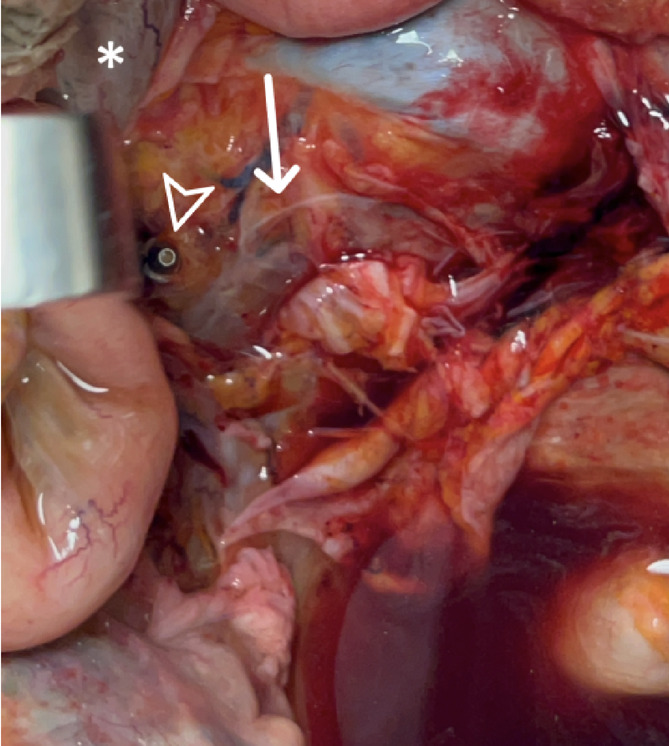
Right pedicle screw tip (arrowhead) abutting the right common iliac vein (asterisk), which is retracted with a Langenbeck retractor. Arrow indicates the promontory.

**Fig. 3:**
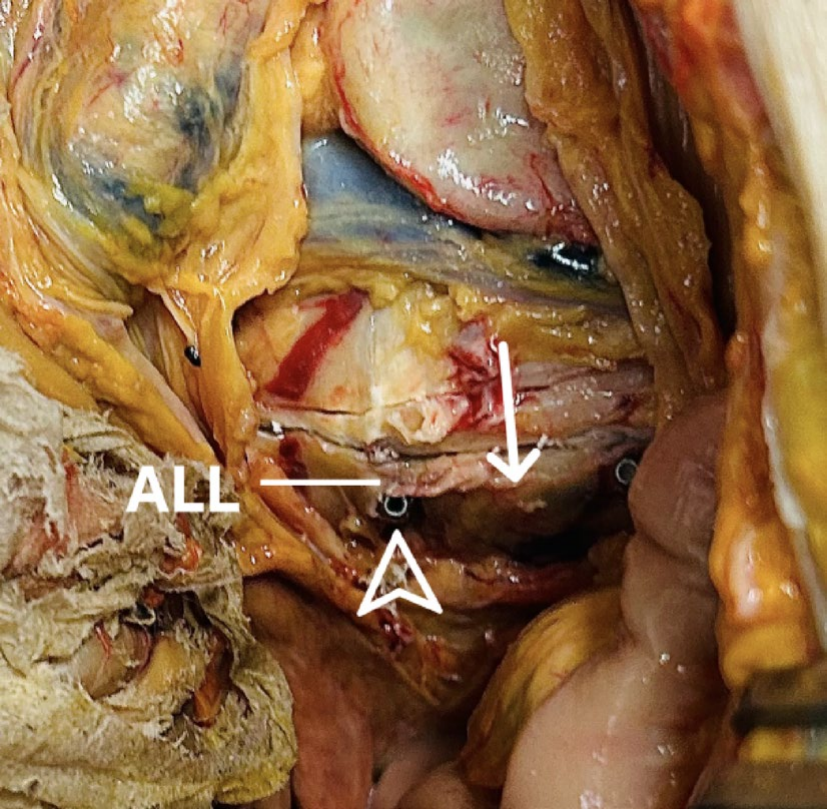
Right S1 pedicle screw tips (arrowhead) penetrating the promontory (arrow). Situs after removal of the anterior longitudinal ligament (ALL). Note that no intrapelvic vessels are compromised. Disc space is incised at the endplates.

None of the pedicle screw tips penetrated into the lumen of a major presacral vessel. In two cases of the 12 dissected specimen (8.3%), the screws abutted the right common iliac vein (CIV) after penetrating the anterior longitudinal ligament (ALL). They were in close contact with the dorsal adventitial layer, but did not cause injury (Fig. 2). In another two cases (8.3%), the right pedicle screw tip was oriented dorsally to the right CIV, but was covered by the ALL. Both structures, the ALL and the CIV remained completely intact. In the remaining specimens (84%), the screw tips were also covered by the ALL, but entirely surrounded by osteophytes (Table 1). Their tips were not directed toward major intrapelvic vessels (Fig. 3).

Seven specimens were imaged using 3D fluoroscopy. Two of these specimens were additionally dissected to confirm the radiological measurements by direct visualization.

As preoperatively planned, the medial convergence of pedicle screw placement measured on selected images ranged from 15° to 21.5° (mean convergence: 18.9°), and each screw exceeded the anterior cortex by two threads.

The mean distance from the screw tip to the iliac vessels, measured on selected images, was 9.4 mm on the right side and 12 mm on the left side, with no evidence of neurovascular conflict.

**Table 1:** Sacral pedicle screw abutment - count.

Screw abutment - dissected specimen			
	No	Yes	Grand Total
Right	10	2	12
Left	12	0	12
Grand total	**22**	**2**	**24**

## Discussion and clinical significance

Fractures or deformities are traditionally stabilized by open surgery. Yet, open surgery often involves large incisions and extensive muscle dissections, which might trigger significant surgical trauma and blood loss. This can cause long recovery periods, postoperative pain, and an increased risk of complications, such as infections^9,10^. An alternative to standard open internal fixation (OIF) of spinal instrumentation is minimal invasive percutaneous surgery fixation with pedicle screws^11^. Various studies, have highlighted the benefits of this technique and described different methods^12^. Especially the outcome of tricortical pedicle screw fixation is convincing. Here the screws are placed to penetrated three cortical layers until their tips penetrate into the apex of the promontory^11,13^. Tricortical screw fixation offers superior stability and reduced loosening compared to monocortical fixation, making it the preferred technique for patients requiring robust spinal constructs, especially at the lumbosacral junction^1,2,13^.

A potential complication of tricortical percutaneous pedicle screw insertion is the penetration of neurovascular structures anterior to the sacrum and lumbosacral junction^4,5,8,14,15^. However, retrospective analyses revealed such complications in only 0.15% of the interventions in freehand technique; or even less^15^. Our anatomic study shows that the CIV is the vessel with the highest risk of being injured. Yet in none of our cases it was perforated or injured. In addition, aligning with the findings of Ergur et al., the bifurcation of the aorta and the inferior vena cava were located cranially to the sacral promontory and thus also remained unharmed in all specimens^4^.

Our study showed, that a mean convergence of 18.9° can be achieved with tricortical percutaneous screw fixation. The screw tip exceeded the anterior cortical layer by two threads. In 4 cases (16.7%) screw trajectory aimed at iliac vessels, of which two screws (8.3%) came into contact with the vessels, but without vascular injury. So in PTSPSF, the trajectory of the S1 screw put 4 cases (16.7%) at risk of vascular injury. Compared to 26.5% in open tricortical fixation and 45.4% in non-tricortical fixation cases^13^ this cadaveric study demonstrates that PTSPSF is a safe procedure.

Although the mean distance from the screw tip to the iliac vessels was approximately 1 cm (12 mm left and 9.4 mm on the right) the risk of vascular injury is primarily associated with trajectories where the screw directly contacts or impinges upon a vessel. Conversely, when the screw trajectory does not intersect the course of a vessel, the risk remains low despite close proximity.

The guide wire should never be pushed anterior to the cortex, where it could injure the iliac veins. It is conceivable that vessel injury could occur with deeply dislocated K-wires when the trajectory of the screw conflicts with the iliac vessels.

We identified the presence of osteophytes near the promontory to contribute to the low incidence of blood vessel injuries. They are not visible during lateral fluoroscopy. However, as our results show, they surround the screw tips, and provide a protective barrier for the pelvic neurovascular structures.

The most critical factor influencing the safety of tricortical S1 fixation is the orientation of the screw trajectory. While the presence of anterior osteophytes and the tensile strength of the anterior longitudinal ligament may provide some protective buffering, they should not be regarded as the primary determinants of safety. Instead, the direction of screw insertion relative to the promontory and adjacent neurovascular structures determines the risk profile and should be emphasized in both surgical training and intraoperative navigation.

In the two cases, where the screws penetrated the ALL and abutted the right CIV, secondary injury due to arterial walls pulsating against instrumentation in vivo might be possible, though the vessels were not deformed in our study.

In our cadaveric series, no acute vascular injuries were observed despite the anterior cortex being penetrated by the tricortical screws. However, in these two cases the screw tips were in close contact with the adventitial layer of the common iliac vein. Although no macroscopic perforations occurred, the possibility of long-term damage due to chronic pulsatile friction or sustained wall compression cannot be excluded. This potential risk underscores the importance of careful trajectory planning.

In this study, the screws used measured 60 mm in length. The screw tip reached two threads beyond the anterior cortex in each case. In close-up observations, this position resulted in mild vessel wall contact in two cases, though without penetration or deformation. These findings suggest that careful limitation of screw length, coupled with strict control of the insertion angle, are essential to minimize the risk of vascular compromise.

Parker et al. found that among 3,443 thoracic pedicle screws placed, 10 screws (0.29%) encroached on major vascular structures. Of these, two asymptomatic patients out of 3,443 (0.06%) underwent revision surgery as the pedicle screws were abutting and deforming the aorta^15^. On the other hand, Foxx et al. detected no case of vessel injury despite finding 11 (1.6%) of 680 screws in contact with arterial vessels on postoperative imaging. So they hypothesized that screws contacting but not penetrating or deforming major vessels can be safely observed^8^.

Although our study uses fresh, randomly selected body donors and thus closely resembles the conditions in patients, our setting has limitations. Firstly, simulation of surgical procedure in cadavers does not fully replicate live surgical conditions in patients, where tissue response, bleeding, and other dynamic factors play a significant role. To minimize the effect of this limitation we used unembalmed specimens, which preserve tissue characteristics and thus are superior to formaldehyde-preserved cadavers. Secondly, a cadaveric study does not permit follow up examinations. Thus the risk of patients to develop secondary vascular injuries, due to chronic erosion of arterial walls pulsating against instrumentation^16,8^ cannot be estimated. Thirdly, body donors usually have died in advanced age. This influences the frequency of osteophytes, as these are generally considered as a degenerative, age-related condition^7,17,18^.

## Conclusions

In our study, we used fresh cadaveric specimens to preserve tissue integrity while inserting tricortical pedicle screws via a minimally invasive percutaneous approach. Our focus was on the protective mechanisms provided by osteophytes and the anterior longitudinal ligament (ALL), offering crucial new insights beyond the general discussion of anatomical risks.

We observed the lowest risk of vascular injury when screws were placed as convergently as possible, aiming for the midpoint of the sacral promontory. However, this optimal trajectory can be limited by the S1 sacral crest. Given our findings, we recommend taking particular care to avoid injury to the common iliac veins, which were the most at-risk structures in our study.

Osteophytes at the promontory—commonly found in patients with degenerative spine disease—along with the ALL, provide a degree of natural protection. However, since these structures are not visible under fluoroscopy and can be compromised by excessive transgression of the promontory cortex, they should not be relied upon as the sole protective barriers.

This study aimed not only to highlight surgical risks but also to identify key anatomical structures that offer protection. Based on our findings, we recommend targeting the midpoint of the promontory, as this approach minimizes the risk of injuring the iliac vessels while maximizing the potential benefit of protective osteophytes. A key technical aspect is that the screw tip should not extend more than two threads anterior to the sacral promontory cortex.

## Data Availability

All data generated or analyzed during this study are included in this published article.
